# Self-perceived competency of midwives in Kenya: A descriptive cross-sectional study

**DOI:** 10.4102/phcfm.v14i1.3477

**Published:** 2022-12-14

**Authors:** Edna C. Tallam, Doreen Kaura, Robert Mash

**Affiliations:** 1 Department of Nursing and Midwifery, Faculty of Medicine and Health Sciences, Stellenbosch University, Cape Town, South Africa; 2 Nursing Council of Kenya, Nairobi, Kenya; 3 Department of Family and Emergency Medicine, Faculty of Medicine and Health Science, Stellenbosch University, Cape Town, South Africa

**Keywords:** midwifery, nurse-midwife, education, competence, competency

## Abstract

**Background:**

Midwifery competence is demonstrated in the context of midwifery education, regulation and practice to support the quality delivery of care to women. Midwives with appropriate competencies can deliver up to 80% of maternal health services. The pre-service education programmes in Kenya offers different midwifery competencies for the various programmes, influencing expected outcomes in practice.

**Aim:**

This study aimed to assess midwives’ perceived level of competence based on the International Confederation of Midwives (ICM) standards in Kenya.

**Setting:**

The study was conducted in selected public health facilities in Kenya.

**Methods:**

An observational cross-sectional design was used. A multi-stage sampling technique was used to select the counties and health facilities and random sampling to determine 576 midwives. Data were collected using a self-administered assessment tool adopted from the ICM competency domains.

**Results:**

A total of 495 (85.9%) midwife respondents participated in this study, of which 389 (78.6%) respondents in all training categories were highly competent in the four ICM domains. The midwives’ qualifications and facility level were associated with their self-perceived competence during practice. Those trained in the direct-entry midwifery programme were more competent, *p* = 0.016 (Kruskal wallis H = 8.432).

**Conclusion:**

Midwives’ competence was influenced by the level of education and facility where they practice. All pre-service midwifery programme graduates must meet the essential ICM competencies and need to enhance continuous professional development (CPD) programmes and facility-based mentorship for the midwives.

**Contribution:**

To optimise midwifery-led practice in primary health care, midwifery competence should be enhanced in pre-service and in-service education for improved health outcomes.

## Introduction

Globally, over 70% of maternal deaths are caused by complications of pregnancy and childbirth and complications of preterm birth account for more than 85% of newborn deaths.^1^ High-quality care to prevent and manage complications during this critical period is likely to reduce maternal deaths, stillbirths and early neonatal deaths significantly.^2^ There has been a progressive global maternal mortality rate improvement.^3^ In Kenya, the maternal mortality rate dropped from 488/100 000 in 2008 to 362/100 000 live births in 2014,^4^ although mothers continue to die from preventable morbidities.^5^ Reducing maternal and neonatal mortality rates and eliminating preventable causes of death require delivery of quality care from preconception to pregnancy, labour and ongoing care of the mother and newborn.^6^

Midwives are integral in providing quality maternal and neonatal health (MNH) care and reducing preventable maternal and perinatal mortality and morbidity. Midwives are the majority providers of MNH, offering over 83% of the care needed by women and newborns.^1^ Thirty-one interventions delivered by a midwife, trained with International Confederation of Midwives (ICM) standards, can potentially prevent 4.3 million maternal deaths, stillbirths and neonatal deaths annually by 2035.^7^ However, the State of the World’s Midwifery Report (2021) indicated that there is a current global shortage of 900 000 midwives, and there is a need for bold investments in the education and training of midwives to meet the essential reproductive, maternal and newborn health needs.^8^ Kenya suffers from this acute shortage of nurses or midwives. The current estimates show a workforce of 100 nurses or midwives per 100 000 population, lower than the World Health Organization (WHO) recommended minimum staffing level of 356 nurses or midwives per 100 000 population.^9^

The ICM has set global standards for minimum essential competencies for midwifery practice to provide high-quality, evidence-based health services for women, newborns and childbearing families.^10^ These competencies are defined as the minimum set of knowledge, skills and professional behaviours required by an individual to use the designation of midwife when entering midwifery practice. The competency framework has four broad domains: general, pre-pregnancy and antenatal care, labour and birth, and ongoing care for the woman and newborn.^10^

Proficiency in these domains is expected to equip midwives with the necessary competencies and confidence to enhance the care of the woman and the neonate. A competent midwife will contribute to achieving the third sustainable development goal (SDG),^11^ which aims to reduce the global maternal mortality ratio to less than 70 per 100 000 live births and neonatal mortality to less than 12 per 1000 live births by 2030.^12^

However, there are inadequacies in midwifery education in low- and middle-income countries. These include deficient curriculum with tutors confident in theoretical teaching and not practical skills teaching with limited exposure to clinical practice for students.^13,14,15^ This may have profound implications on producing competent midwives who can provide the full range of services needed.^16^ Midwifery education and training in Kenya and the East Africa region has been a subset of the nursing education model, including certificate, diploma and degree qualifications. Gaps in midwifery practice may be related to these different qualifications, which have led to poor alignment of the required competencies as defined by the ICM, the scope of practice defined by regulators in other countries and the learning outcomes of various educational programmes within those countries.^17,18^

Because of the overwhelming need for midwifery services in primary health care, midwives often function independently and take responsibility for clinical decisions and management of women and newborns. Therefore, enhancing their competencies and practice within their scope of practice is essential as an autonomous profession.

Confidence and competence of a midwife are required for optimal performance of a task.^16^

Evidence from developed countries showed low levels of competence among graduate nurses in legal and ethical practice and advocated for support to enhance the competence as they enter practice.^19^ In Sweden, newly qualified midwives were reported to lack the experience, which affected their competence.^20^ In China, midwives reported higher self-perceived essential competencies in the provision of vaginal delivery with education levels and years of experience affecting their competencies.^19^ Even though there is evidence on the competence of midwives in their scope of practice in developed countries, there is limited evidence on the same in developing countries including Kenya.

In Kenya, a national confidential enquiry into maternal deaths (CEMD) in 2017 revealed that 92% of mothers received suboptimal care from skilled health personnel, with midwives providing most of the care. Inadequate clinical skills, inadequate monitoring and lack of timely intervention for mothers with prolonged abnormal observations were identified as the main contributing factors to the maternal deaths.^5^ This points to the challenges with competence levels of the midwives who are expected to function autonomously within the ICM and national scope of practice. Therefore, further evaluation needs to be conducted on midwives’ competencies in practice as per the ICM standards. Therefore, this study aimed to assess the perceived level of competence among midwives in practice with different midwifery qualifications in Kenya.

## Research methods and design

### Study design

This article reports on a component of phase 1 of an explanatory, sequential, mixed-methods study. Phase 1 of the study was a quantitative, observational, descriptive, cross-sectional study design focused on self-perceived competence and confidence of midwives. The other quantitative component in phase 1 on self-perceived confidence of midwives on knowledge and skills has already been published.^21^ Phase 2 of the study is planned to qualitatively explore and explain the findings of phase 1 studies through descriptive phenomenological design.

### Setting

Health care in Kenya is offered at primary (dispensaries and health centres), secondary (sub-county and county referral hospitals) or tertiary (national teaching and referral hospitals) levels.^9^ This study was conducted in public secondary and tertiary health care facilities because the departments or units in these institutions that offer the comprehensive reproductive, maternal and newborn child health (RMNCH) services are distinct in that the midwives deployed in these units were the study participants. Furthermore, most primary health facilities do not offer the comprehensive RMNCH services, and nurse midwives are providing varied services across all units in the facility.

Private and faith-based facilities were excluded, as midwives could not always practise autonomously in these settings. Midwives in Kenya train and qualify at certificate, diploma, higher diploma or degree (bachelor, master or doctoral) levels and are licensed to function at all levels of health care facilities. Common midwife cadres in Kenya include Kenya Registered Midwifery (KRM), Kenya Registered Community Health Nursing (KRCHN) and Bachelor of Science of Nursing (BScN). The KRCHN and BScN programmes are integrated and train nurses or midwives who qualify with general nursing, midwifery, mental health and community health competencies. The KRM programme only trains students who are to be midwives.

### Study population and sampling strategy

The study population included midwives working in public secondary or tertiary health facilities. As of 2019, Kenya had 20 775 registered midwives distributed by qualifications: 432 KRMs, 19 477 KRCHNs and 866 BScNs.^22^

### Sample size determination

The sample size was calculated for midwives with each qualification to ensure precise competence estimation across all programmes. According to a similar study in Ethiopia, 31.6% of midwives attained the minimum required competency score, and this proportion was used in the sample size calculation.^23^ Kenya and Ethiopia are both in Eastern Africa and share similar challenges, but there is no such study conducted in Kenya. The sample size calculation also assumed a confidence interval of 95%, a margin of error of 5% and a design effect of 1.0. The sample size was also corrected for finite population size, the final representative sample size was 576, with 82 (8.8%) KRMs, 295 (59.6%) KRCHNs and 199 (31.5%) BScNs.

### Sampling technique

**FIGURE 1:**
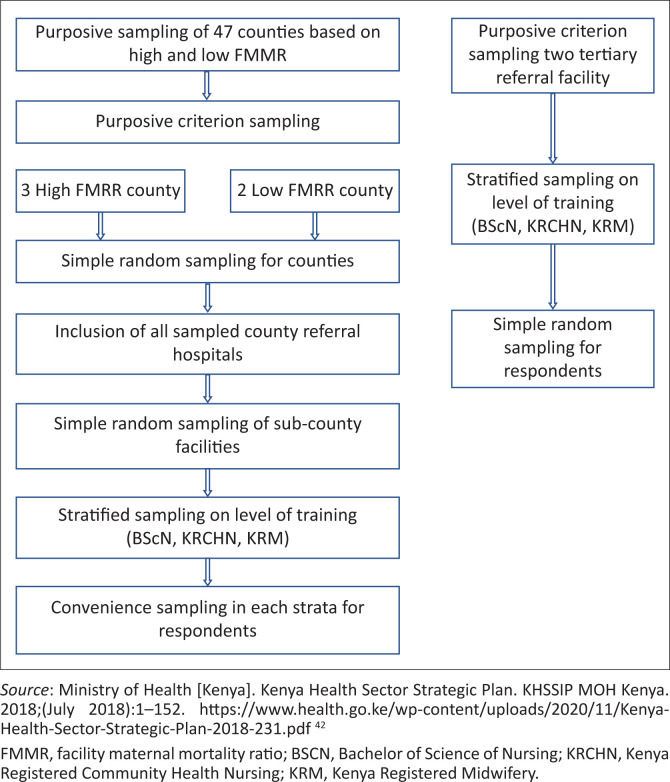
Multi-stage sampling framework.

A multi-stage stratified sampling approach was used, as shown in Figure 1. The sample was split between the secondary levels of care (in the counties) and tertiary hospitals based on the number of midwives who worked at RMNCH units. The sample needed from the counties was 301 (52.3%) and 275 (47.7%) from the tertiary hospitals.

The 47 counties were divided into two groups based on their facility maternal mortality ratio (FMMR). Kenya considers an average FMMR of 97.5 as a threshold for high versus low performing facilities.^4^ Twenty-one counties out of 47 had a high FMMR > 97.5, while 26 counties had a low FMMR of < 97.5. Four counties were then randomly selected from each group (four with a high FMMR and four with a low FMMR).

All eight county referral hospitals were included in the study. Moreover, three sub-county facilities were randomly sampled from each of the eight counties. In total, therefore, the study sampled 32 facilities in the counties. The required sample of 301 from these 32 facilities was further stratified by the required qualifications – KRMs (54), KRCHN (137), BScN (110) – and the distribution of midwives between the facilities.

The tertiary hospital sample of 275 was stratified between the two hospitals based on the proportion of midwives employed; this implied a sample of 167 midwives from tertiary hospital A and 108 from tertiary hospital B. Stratification was then done according to the proportion needed with each qualification category KRM (27), KRCHN (158) and BScN (89) based on the distribution of midwives in the two facilities.

### Data collection

The study was conducted between November 2019 and March 2020. The questionnaire was adapted from the 2019 ICM self-assessment tool, which assessed the level of competence and confidence in knowledge, skills and behaviour in the four ICM domains. Skills were defined as the ability to perform a specific task to a measurable level of performance, while behaviour was defined as a person’s way of responding to the actions of others.^10^ The first domain (general competency) had 13 items, the second domain (pre-pregnancy and antenatal care) had nine items, the third domain (care during labour and birth) had two items and the fourth domain (ongoing care of the woman and newborn) had five items.

Nine experts’ panellists evaluated the questionnaire’s content validity. They were either working in the facilities as midwives, in policy or educators in midwifery in Kenya. The investigator requested the experts to independently review and critique each item to ensure the appropriateness of the content and phrasing of each item using a three-point Likert scale (3, essential; 2, useful; and 1, not necessary). The content validity ratio (CVR) was calculated for each item and a ratio of 0.8 or more was taken as acceptable.^24^

The panel of experts adapted the tool and modifications were done in terms of the content, the phrasing of the items, the instructions for completing the questionnaire, layout, formatting and context-specific demographic data. Two items on ongoing care of the woman, which the experts indicated were inappropriate for the Kenyan context, were deleted. These items were related to the religious, cultural and socio-political environment. The removal of the two items was done before the pilot study.

A pilot study with 50 participants (10% of the sample size) further validated the questionnaire and checked the data collection process. From the pilot study, time allocated to the study was adjusted from 30 min to 45–60 min; paraphrasing some sentences and revising the instructions for filling the tool were also incorporated. These participants were from a different county, not part of the study. The pilot data were tested for reliability, and an adequate average Cronbach’s alpha coefficient of 0.87 was achieved in all four competency domains of the tool.^25^

Research assistants (RAs) were recruited from other facilities, not part of the study. The assistants did not hold any managerial positions to minimise any power imbalance with the participants. The participant information sheet and consent form were given in hardcopy and electronically, 48 h before the start of data collection. Once the participants signed the consent form, a copy was provided. A convenient time to complete the questionnaire was agreed upon in order to not disrupt the service delivery. The research team addressed any questions on the data collection day. This was an interviewer-administered questionnaire, where the RAs administered the questionnaire to each participant in a private room at the hospital. The RA interviewed each of the participants, which took about 45–60 min and captured the data in real time using organisation network analysis (ONA) software during the interview.

### Data management and analysis

Data were checked continuously during data collection, and any inconsistencies or incomplete data were corrected by following up with the respondents. Data captured in the ONA software were exported to the Statistical Package for Social Sciences (SPSS) version 26.0 for analysis.

Competence was self-rated on two dimensions, namely, knowledge and skills. Knowledge was rated according to how long ago the midwife updated her knowledge through short courses, seminars, conferences or workshops. The knowledge scale was defined as current knowledge (updated within 1 year, high score 3), updated knowledge within the last 2–3 years (moderate score 2) and updated knowledge > 3 years ago (low score 1).

The midwife’s skills were assessed according to the midwife’s self-perceived safe performance of the skill (i.e. no preventable complications and with good maternal and neonatal outcomes). Their skills were rated according to how often they were safely performed, and the scale was defined as follows: not performed skill safely within the past year (low score of 1), performed skill only once within the year (moderate score of 2) and performed skill more than once within the year (high score 3).

As defined by ICM, a holistic assessment of competence included up-to-date knowledge and experience of performing the skill.^10^ A competence mean score was calculated for each item based on the sum of the knowledge and skill score. The competence mean score was then interpreted as 1–1.49 (low competence, ranked as 1), 1.5–2.49 (moderate competence, ranked as 2) and 2.5–3.0 (high competence, ranked as 3).

Categorical data were summarised using frequencies and percentages. In contrast, continuous (numerical) variables were summarised using means or medians with standard deviation (s.d.) or interquartile ranges (IQR) depending on the distribution of the data.

The association between competence levels and socio-demographic characteristics, facility level and qualifications was analysed using the Pearson’s chi-square test (Fisher’s exact test where cells had counts less than 5). The difference between the dependent variable (competence levels for knowledge and skills) and the independent variables (socio-demographic characteristics, the level of the facility and midwives’ qualifications) was analysed using the Kruskal–Wallis test. Post hoc analysis was performed where there was a statistically significant difference in categorical variables. All statistical tests were tested at a 5% significance level, and a *p*-value of < 0.05 was considered statistically significant.

### Ethical considerations

Ethical approval to conduct the study was obtained from the following institutions: Stellenbosch University Health and Research Ethics Committee 2 (HREC2) (No. S18/10/254); AMREF Ethics and Scientific Review Committee (ESRC) (No. AMREF – ESRC P 652/2019); Moi Teaching and Referral Hospital-Moi University Institutional Research and Ethics Committee (IREC) (No. IREC/2019/000092) and Kenyatta National Hospital-University of Nairobi Ethics and Research Committee (ERC) (No. KNH-ERC/A/73). Research license was obtained from the National Commission for Science, Technology and Innovation (NACOSTI) (No. 8777616). Permission was also sought from the county government, while the respondents gave informed consent.

## Results

### Characteristics of respondents

A total of 495 (representing 86% of the required sample size) midwives completed the questionnaire. The distribution of the midwives by qualifications was as follows: those with KRCHN – 295 (100% of the required sample size), BScN degree – 156 (78%) and those with the KRM diploma – 44 (54%).

The characteristics of the midwives are presented in Table 1. Data were collected in 26 facilities: 2 tertiary, 6 county and 18 sub-county facilities. Midwives were spread between levels of care, with 39.6% at the tertiary level, 28.7% at the county level and 31.7% at the sub-county level (*p* = 0.714). Female midwives accounted for 391 (79.0 %), with almost half of the respondents (237; 47.9 %) aged between 30 and 39 years, with a median age of 37.0 years (IQR 32.0–43.0).

**TABLE 1:** Characteristics of respondents.

Characteristics	KRM (*N* = 44)		KRCHN (*N* = 295)		BScN (*N* = 156)		Total (*N* = 495)		*p*
	*n*	%	*n*	%	*n*	%	*n*	%	
**Facility levels**									
Tertiary hospitals	15	34.1	108	36.6	73	46.8	196	39.6	0.174
County hospitals	15	34.1	84	28.5	43	27.6	142	28.7	-
Sub-county facilities	14	31.8	103	34.9	40	25.6	157	31.7	-
**Age**									
< 30 years	4	9.1	54	18.3	19	12.2	77	15.6	0.015
30–39 years	17	38.6	132	44.7	88	56.4	237	47.9	-
40–49 years	14	31.8	87	29.5	40	25.6	141	28.5	-
≥ 50 years	9	20.5	22	7.5	9	5.8	40	8.1	-
**Gender**									
Female	36	81.8	235	79.7	120	76.9	391	79.0	0.707
Male	8	18.2	60	20.3	36	23.1	104	21.0	-
**Years of work experience**									
< 5 years	8	18.2	70	23.7	25	16.0	103	20.8	0.001
5–9 years	6	13.6	106	35.9	51	32.7	163	32.9	-
10–14 years	10	22.7	40	13.6	41	26.3	91	18.4	-
15–19 years	9	20.5	32	10.8	23	14.7	64	12.9	-
≥ 20 years	11	25.0	47	15.9	16	10.3	74	14.9	-
**Facility maternal mortality**									
Low	11	25.0	96	32.5	36	23.1	143	28.9	0.090
High	33	75.0	199	67.5	120	76.9	352	71.1	-

KRM, Kenya Registered Midwifery; KRCHN, Kenya Registered Community Health Nursing; BScN, Bachelor of Science of Nursing.

Midwives from the KRM programme (median age 40.0 years [IQR 32.5–47.0]) were significantly older than those from the KRCHN (median age 36.0 years [IQR 30.0–43.0]) and BScN programmes (median age 37.0 years [IQR 32.0–43.0]) (*p* = 0.04). Midwives differed significantly in their years of experience per qualification, from the least experienced with KRCHN (median experience 7.0 years [IQR 5.0–15.0]) to BScN (median experience 10.0 years [IQR 5.5–14.5]) to the most experienced with KRM (median experience 13.0 years [IQR 6.5–19.5]) (*p* = 0.007). There was no significant association between FMMR of facilities or gender with the type of qualification.

### Self-perceived knowledge and skills

The overall levels of self-perceived knowledge and skills for midwives with different qualifications are presented in Table 2. Overall, the majority of the midwives reported to be up to date with their knowledge (378; 76.4%) and could safely perform the required skills (406; 82.0%).

**TABLE 2:** Self-perceived knowledge and skills.

Characteristics	Midwives’ qualifications								*p*
	KRM		KRCHN		BScN		Total		
	*n*	%	*n*	%	*n*	%	*n*	%	
**Knowledge status**									
Low	0	0.0	5	1.7	0	0.0	5	1	0.019
Moderate	8	18.2	68	23.1	36	23.1	112	22.6	-
High	36	81.8	222	75.3	120	76.9	378	76.4	-
**Level of skills**									
Low	0	0.0	3	1.0	1	0.6	4	0.8	0.016
Moderate	3	6.8	51	17.3	31	19.9	85	17.2	-
High	41	93.2	241	81.7	124	79.5	406	82.0	-

KRM, Kenya Registered Midwifery; KRCHN, Kenya Registered Community Health Nursing; BScN, Bachelor of Science of Nursing.

There were, however, significant differences between qualifications in terms of knowledge status (*p* = 0.09) and safe performance of skills (*p* = 0.016). For instance, KRM-qualified midwives had higher knowledge status (81.8%) as compared to KRCHN and BScNs.

For knowledge status, direct-entry midwives at the diploma level (KRM) reported higher levels of knowledge (36; 81.8%) compared to KRCHNs and BSNs, respectively. Similarly, the KRMs had higher ratings of 41 (93.2%) for safe performance of skills compared to KRCHNs and BScNs.

### Respondents’ competence status

Table 3 presents the proportion of midwives by competence status. Overall, 389 (78.6%) of the midwives reported high competence across all the ICM domains. Midwives reported the lowest level of competency in the general competency domain (64.4% had high competence), followed by pre-pregnancy and antenatal care (76.6%), ongoing care (81.2%) and finally labour and childbirth (82.0%). Only a small proportion of less than 1% of the midwives reported low competence in the four ICM domains.

**TABLE 3:** Proportion of respondents by competence status (*N* = 495).

ICM competency domains	Low competence		Moderate competence		High competence	
	*n*	%	*n*	%	*n*	%
General competency	4	0.8	172	34.7	319	64.4
Pre-pregnancy and antenatal	5	1.0	110	22.2	380	76.6
Labour and birth	4	0.8	85	17.2	406	82.0
Ongoing care of woman and baby	2	0.4	91	18.4	402	81.2
Overall competence	3	0.6	103	20.8	389	78.6

ICM, International Confederation of Midwives.

The association between competence, socio-demographic characteristics and FMMR is presented in Table 4. There was no significant association between competence levels and age, gender, FMMR or work experience.

**TABLE 4:** Association between competence and socio-demographic or facility maternal mortality ratios.

Characteristics	Low competence (*N* = 3)				Moderate competence (*N* = 103)				High competence (*N* = 389)				Total (*N* = 495)				*p*
	*n*	%	Median	IQR	*n*	%	Median	IQR	*n*	%	Median	IQR	*n*	%	Median	IQR	
**Facility maternal mortality ratio**																	
Low	2	1.4	-	-	26	18.2	-	-	115	80.4	-	-	143	100	-	-	-
High	1	0.3	-	-	77	21.9	-	-	274	77.8	-	-	352	100	-	-	0.207
**Gender**																	
Female	3	0.8	-	-	86	22.0	-	-	302	77.2	-	-	391	100	-	-	0.332
Male	0	0.0	-	-	17	16.3	-	-	87	83.7	-	-	104	100	-	-	-
Age of the respondent	-	-	32.0	30.0, 42.0	-	-	38.0	32.0, 45.0	-	-	36.0	32.0, 42.0	-	-	37.0	32.0, 43.0	0.229
Work experience	-	-	7.0	4.0, 7.0	-	-	10.0	5.0, 18.0	-	-	8.0	5.0, 15.0	-	-	9.0	5.0, 15.0	0.140

IQR, interquartile ranges.

### Relationship between qualifications and competence

**FIGURE 2:**
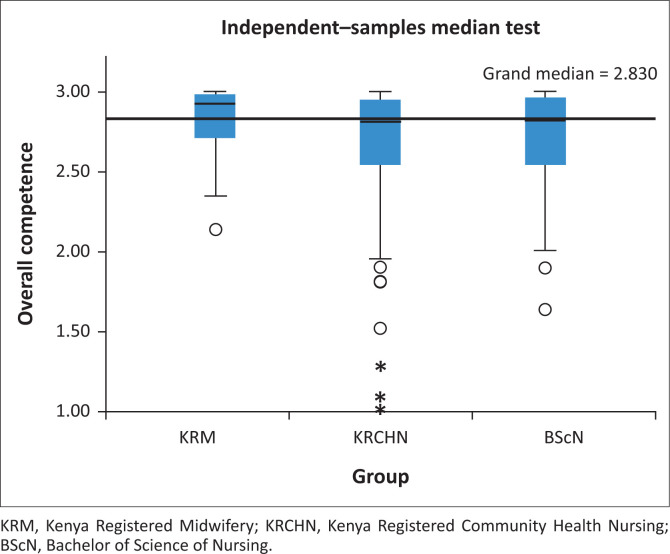
Association between competence and qualifications levels.

The relationship between qualifications and competence is shown in Figure 2. The median overall competence score for KRM was 2.93 (IQR 2.4–3.0), for KRCHN was 2.80 (IQR 1.9–3.0) and for BScN was 2.82 (IQR 2.0–3.0) (*p* = 0.016). Post hoc analysis demonstrated that statistical significance was because of the difference between KRM and other qualifications (KRM-KRCHN [*p* = 0.004]; KRM-BScN [*p* = 0.018] KRCHN-BScN [*p* = 0.549]).

### Relationship between facility level and competence

**FIGURE 3:**
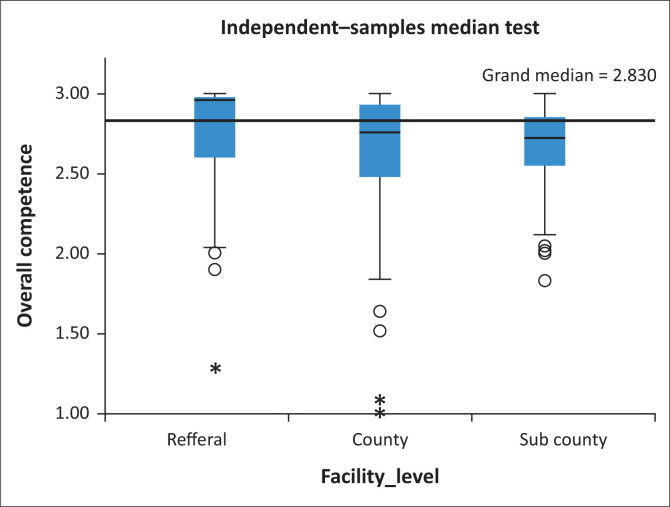
Association between competence and facility levels.

The relationship between competency and facility level is shown in Figure 3. The median overall competency score for the tertiary referral level was 2.98 (IQR 2.0–3.0), for the county level was 2.80 (IQR 1.7–3.0) and for the sub-county level was 2.82 (IQR 2.2–2.8) (< 0.001). Post hoc analysis demonstrated that statistical significance was because of the difference between the tertiary referral hospitals and those who were based at county and sub-county facilities (sub-county-county, *p* = 0.317; sub-county-tertiary referral, *p* < 0.001, county-tertiary referral *p* < 0.001).

## Discussion

Most of the midwives were highly competent in their competence, knowledge and skills. This implies that only 21% of midwives were not highly competent. The type of qualification and facility level were associated with the self-perceived competence of midwives in Kenya. Those who qualified as direct midwives had a higher median score than those who qualified as nurse midwives at the diploma and degree levels. Moreover, those who worked at the tertiary level had a higher score than those at the county and sub-county health facilities. The findings of this study are similar to studies conducted in India and Tanzania where nurse midwives from lower-level facilities had low competence and/or skills levels compared to those from higher facilities.^26,27^ Therefore, clients visiting lower-level facilities cannot be considered to have access to competent midwifery care. This calls for urgent measures to effectively increase the competence of the midwives working in these settings to contribute to the access of quality universal health coverage and reductions in maternal and newborn mortalities in Kenya.

Globally, midwives are expected to update their knowledge and skills for safer practice and improved maternal and newborn health outcomes through continuous professional development (CPD) programmes.^10,28^ Other external factors, such as learning environment, quality assurance systems and mentoring, support the acquisition of knowledge and overall competence. In-service midwifery education and training enable learners to update and enhance their knowledge and skills in practice.^23,29^

The KRCHN and BScN midwives had similar competency scores but were significantly lower than the direct-entry KRM midwives. The KRCHN and BScN programmes strongly focus on nursing as a whole; thus, graduates are likely to have less exposure to midwifery competencies. Similarly, the finding of the present study is supported by a study in Pakistan, which found that direct entry to midwifery education positively influenced graduates’ perceptions of their knowledge, skills and behaviours and their practice.^30^ These findings underscore the need to review and align the training curricula for all midwives with ICM competencies to meet MNH targets.^15,31^ This is consistent with the East Africa Countries (EAC) harmonisation report that recommended direct-entry midwifery training and need to review current blended nurse-midwifery programmes to enhance midwifery competencies in pre-service education.^17^

Compared to county and sub-county levels, tertiary referral facilities have more expertise and specialists, who function in multidisciplinary teams. They have more resources, opportunities and structured systems for CPD that support knowledge and skills development. Regular exposure to performing skills in practice over time influences a midwife’s competence.^32^ However, this is a challenge at the lower-level facilities where midwives are expected to be more independent practitioners. World Health Organization and ICM assert that midwives must be competent to provide optimal quality of care to women and their newborns.^33^ A study in China on midwives’ competencies demonstrated that those who had CPD often were highly competent.^34^

Although a trained midwife is assumed to have the requisite knowledge and skills for safe practice, this study showed that only 64% of midwives had a high level of competence in the general competency domain (ICM Competency 1); this domain assessed the autonomy of a midwife to practise, scope of practice and communications skills and prevention of health problems of reproduction and early life, which are fundamental in promoting the health and overall well-being of the woman and infant.^35^ There is a need for particular focus for CPD in this competency and review the midwifery curriculum to incorporate the domains of general competency.

In the current study, the age of respondents, years of work experience, gender and FMMR did not influence the level of competence. Other studies have found a relationship between gender, age and the years of experience with competence.^23,36,37^ This implies that other factors influencing competence, such as the work environment, types of training programme and different levels of care, may have been more important in enhancing the competence among the midwives.

### Strengths and limitations of the study

To the best of our knowledge, this is the first study to assess the perceived level of competence among midwives in practice in Kenya. These findings and the action plan for mitigating against the low competency levels reported among midwives can be generalised to many African settings and other LMICs that suffer from similar challenges in midwifery training, midwifery workforce composition, distribution and shortage as reported in the State of the Midwifery Report 2021 that make achieving universal health coverage elusive.^8^ This study also had some limitations. For instance, the study assessed perceived rather than actual competencies, which is an indirect measure of competence. The authors acknowledge that illusory superiority is a major limitation for studies involving participants self-rating their competencies and abilities as participants are likely to over-rate their actual competencies, abilities and qualities. To minimise this, the study team emphasised the benefits of participating in the study and that the responses provided by the participants were strictly confidential and anonymous. Moreover, the responses were not to be shared with their hospital managers nor did participation in the study form a part of the annual performance appraisal of the staff members during the study participants’ recruitment stage.

### Implications

Our findings revealed that the integrated nurse-midwifery degree and diploma programmes produced less competent midwives compared to direct-entry midwifery programme because their training is less focused on these specific skills set in midwifery competencies. This implies that the majority of the current workforce who provide midwifery care may not meet the competencies to practise as midwives. There is a need to expand the direct entry midwifery training at diploma and degree levels to ensure the adherence of ICM set standards.

Furthermore, the KRCHN and BScN training programmes should pay attention to local context and tailor midwifery-led practice education standards through curricula reviews to enhance midwifery competencies. Moreover, the graduates of these programmes in practice (in-service) should have more targeted CPD to ensure that their competencies in midwifery are maintained, especially in the general competency domain. Policymakers should incorporate the midwifery practitioners and educators in the policy formulation and implementation to address the gaps in training and practice.

Midwives working in tertiary facilities were more competent than those working in county and sub-county facilities. There is a need to invest in midwifery knowledge and skills acquisition among the midwives working in the county and primary level facilities through access to focused CPD programmes for competency enhancement. Moreover, low-dose high-frequency facility-based mentoring of midwives in the lower-level facilities by experienced midwives and skilled health personnel from higher facilities are effective in improving the midwives’ competencies in the provision of quality midwifery care contributing to a reduction in perinatal and maternal mortalities.^38^ Evidence from Tanzania has shown that facility-based capacity strengthening interventions effectively improve the quality of care and maternal and perinatal outcomes in resource-limited settings.^39,40^

Furthermore, there is a need for the health institutions and managers to provide support systems to the midwives in maintaining their competence in practice and strive to change the organisational culture to ensure that the health facilities are learning institutions through facility-based mentoring.^41^ A multi-disciplinary team composed of family physicians, obstetricians and midwives’ experts working in tertiary level facilities should provide mentorship and outreach programmes at county, sub-county and primary level facilities to support the professional development of the midwives for the achievement of the MNH SDGs. Also, there should be a staff rotation programme to the tertiary level of care to reduce the disparity and inequalities in the exposure of skills and knowledge.

A follow-up study should measure the actual competence of midwives and verify the findings of this study. This will provide further insights into current midwifery competence and contribute to a larger body of evidence that can guide midwifery education and training policy in Kenya.

## Conclusion

Overall, 79% of midwives self-reported to have high competence. Graduates from direct-entry midwifery training programmes were more competent than those from integrated nurse-midwifery training programmes. Midwives working at the tertiary hospitals were more competent than those in the county and sub-county levels. All pre-service training programmes must ensure that graduates meet the basic ICM competencies. In-service CPD programmes must focus on midwives in the lower-level facilities and integrated nurse-midwife practitioners.
